# Meat Production from Dairy Breed Lambs Due to Slaughter Age and Feeding Plan Based on Wheat Bran

**DOI:** 10.3390/ani9110892

**Published:** 2019-11-01

**Authors:** Antonino Di Grigoli, Adriana Bonanno, Mansour Rabie Ashkezary, Barbara Laddomada, Marco Alabiso, Francesca Vitale, Francesca Mazza, Giuseppe Maniaci, Paolo Ruisi, Giuseppe Di Miceli

**Affiliations:** 1 Department of Agricultural, Food and Forest Sciences (SAAF), University of Palermo, Viale delle Scienze, 90128 Palermo, Italy; antonino.digrigoli@unipa.it (A.D.G.); mansour.rabieashkezary@unipa.it (M.R.A.); marco.alabiso@unipa.it (M.A.); francy.vitale88@hotmail.it (F.V.); francesca.mazza@unipa.it (F.M.); giuseppe.maniaci@unipa.it (G.M.); paolo.ruisi@unipa.it (P.R.); giuseppe.dimiceli@unipa.it (G.D.M.); 2 Institute of Sciences of Food Production (ISPA), National Research Council (CNR), Via Monteroni, 73100 Lecce, Italy; barbara.laddomada@ispa.cnr.it

**Keywords:** feed restriction, durum wheat bran, growth, carcass, lamb meat, polyphenols, ferulic acid, antioxidant capacity, fatty acids

## Abstract

**Simple Summary:**

The sheep meat sector in southern Italy, based mainly on light milk-fed lambs, requires technical innovations to improve the production system, the product quality, and enhance the consumption of lamb meat. To fulfill these requirements, this investigation aimed to implement feeding strategies to reduce the cost and energy level of diets for dairy breed lambs slaughtered at an older age than the light lambs, applying a feed restriction at 75% and/or including an inexpensive and local byproduct, such as durum wheat bran (DWB), as a fiber source. The proposed feeding plans were suitable to increase the slaughter age of lambs up to 120 days and produce lean carcasses that, compared to those from 90-day-old lambs, were heavier and with improved meat quality in terms of major water retention and tenderness. The dietary inclusion of DWB limited the fat content and improved the health properties of lamb meat with regard to its antioxidant capacity and fatty acid profile, whereas it reduced lambs’ growth when associated with feed restriction.

**Abstract:**

This experiment aimed to investigate the possibility to increase the carcass weight of dairy breed lambs and produce moderate-fat meat by applying inexpensive feeding strategies based on restriction and through the use of a fibrous byproduct such as the durum wheat bran (DWB). Sixty-five 45-day-old lambs of the Valle del Belice breed, divided into 6 groups, were fed alfalfa hay supplemented with concentrate feeds including DWB at 0% or 20% (DWB0, DWB20), supplied ad libitum (L) or restricted at 75% (R), and slaughtered at 90 or 120 days of age. The groups were as follows: DWB0-90L (n = 14), DWB20-90L (n = 14), DWB0-120R (n = 10), DWB20-120R (n = 9), DWB0-120L (n = 9), DWB20-120L (n = 9). The diet did not affect feed intake, growth or carcass weight of lambs fed ad libitum, whereas 120-day-old lambs fed DWB associated to restriction showed the lowest weight gain (105 vs. 170, 185 and 190 g/day in DWD20-120R, DWB0-120R, DWB0-120L and DWB20-120L; *p* = 0.04). The incidence of fat tissue in the hind leg increased (*p* < 0.0001) from 90L (5.82 and 5.45% with DWB0 and DWB20) to 120R (8.80 and 8.43% with DWB0 and DWB20) and 120L lambs (10.7 and 11.8% with DWB0 and DWB20). Older lambs’ meat, compared to that of 90L lambs, showed analogous levels of intramuscular fat, higher water retention, tenderness and lightness, and a more intense red colour. In meat from 120-day-old lambs, DWB intake tended to reduce the fat level (*p* = 0.009) and increased polyphenol content (1.10 vs. 1.62, and 1.02 vs. 1.65 g GAE/kg dry matter (DM) in 120R and 120L lambs; *p* = 0.02), antioxidant capacity (12.8 vs. 14.9, and 12.8 vs. 15.7 mmol trolox eq/kg DM in 120R and 120L lambs; *p* = 0.02), and the presence of n-3 polyunsaturated fatty acids (FA) (1.61 vs. 2.81, and 1.43 vs. 2.61 g/100 g FA in 120R and 120L lambs; *p* = 0.007), thereby improving the meat’s health properties. The panelists perceived the effects of DWB inclusion as well as the feeding level with triangle tests.

## 1. Introduction

The development of efficient and productive livestock farming systems implies the introduction of innovations related to processes and/or products. The sector of sheep meat in southern Italy currently needs to be renewed with regard to both the production system and the product type. Indeed, in this area, as in other Mediterranean countries, sheep meat is obtained mainly from carcasses lighter than 7 kg of milk-fed lambs of dairy breeds slaughtered at about 30 days of age [1,2,3], and is offered with a certain seasonality [4] and fragmentation, often without qualitative reference standards, aspects that weaken its market position. Recently, many consumers have shown appreciation for lamb meat from heavier carcasses [3], and at the same time, required low-fat meat to prevent obesity, atherosclerosis and cardiovascular disease [2].

In this context, the production of lamb meat and the relative consumption could be enhanced by implementing production models that allow the slaughter age to be raised to obtain heavier and more muscled carcasses than those of traditional light lambs and meet the consumers’ preferences with regard to meat quality.

Several studies have indicated that heavier lamb carcasses show a better conformation [5], and higher dressing percentage [3] and meat: bone ratio [6], thus giving a superior meat yield. However, lamb meat from heavier carcasses is characterized by a higher lipid content [7] due to the increased intramuscular fat deposition, which can have negative effects on meat quality with regard to sensory attributes [8] and health properties related to fatty acid profile [9].

However, findings from other authors [10,11] have confirmed the possibility of modulating the post-weaning feeding of lambs of dairy breeds in confined systems and producing heavier carcasses than those of milk-fed lambs without negative effects on carcass traits and meat quality and also reducing the meat fatness at levels appreciable by consumers [12,13].

The feeding regime is definitely one of the aspects to be carefully modulated on order to achieve an acceptable meat quality in terms of fatness and preserve adequate levels of technical-economic efficiency of the production system. In particular, diets designed for lambs of dairy breeds slaughtered at an older age to obtain heavier carcasses than those of light suckling lambs should be able to reduce feeding costs, limit fat deposition, and ensure appreciable nutritional and organoleptic properties for consumers.

Therefore, in order to reduce feeding costs, this experiment proposed the use of one of the main byproducts in the milling industry, durum wheat bran (DWB). This byproduct is cheap and easily available in southern Italy because durum wheat is a staple crop in Mediterranean regions, where is used for high-quality end products such as pasta, couscous and bourghul. While grain protein content, color and gluten strength are considered the most important features needed for use in pasta and bread production [14], DWB shows a high content of fiber and phenolic compounds, in particular ferulic acid with remarkable antioxidant properties [15,16,17,18,19]. The presence of polyphenols in the diet is undoubtedly advantageous to improving the oxidative state and hence the well-being of the animals [20,21]. In this regard, Wang et al. [22] showed that a dietary supplementation of 80 mg/kg of ferulic acid was able to decrease the oxidative stress of lambs in cold environment and improve their growth performance. Moreover, the transfer of antioxidant molecules, such as ferulic acid, could improve the safety, oxidative stability and health properties of animal products, with benefits for consumers.

Accordingly, the present study aimed to investigate the possibility of raising the carcass weight of lambs of dairy breeds and producing medium-fat meat by increasing the slaughter age and applying inexpensive feeding strategies based on restriction and the use of a fibrous byproduct such as DWB.

## 2. Materials and Methods

### 2.1. Lambs, Diets and Experimental Design

The experiment was carried out in the period April–June at the private Bulfara farm, located in Alimena, in the province of Palermo, Sicily, Italy (37°42’ N, 14°08’ E, 650 m above sea level). At weaning, 68 lambs of the Valle del Belice breed, aged about 35 days, were individuated and assigned to two homogeneous groups according to sex, type of birth (single and twin), age and live weight.

Both groups were divided into two subgroups of 17 lambs. Each of these four subgroups was transferred in an indoor straw-bedded pen equipped with two linear feeders of 3.7 m, with access to an outdoor paddock where a water trough was placed. There, the groups were fed immediately with the experimental diets and housed until the lambs were 90 days old.

The experimental period started after 10 days of adaptation to the new housing conditions and feeding treatments, when the lambs averaged 44 ± 6 days of age and 16.2 ± 2.7 kg of live weight.

The experimental diets, fed ad libitum (L), included alfalfa pelleted hay as a common forage basis, supplemented with one of two isoenergetic concentrates consisting of faba bean and barley grains in the form of a coarsely ground meal, and differing in the presence of DWB at 0% or 20% (DWB0, DWB20) (Table 1). Both hay and concentrates were divided into two daily meals, supplied separately in the morning and in the afternoon. 

After 45 days of ad libitum feeding, 7 lambs homogeneously selected from each subgroup, corresponding to 14 lambs for each experimental diet, were slaughtered at an average age of 90 days (90L). In contrast, for 30 days until slaughter at 120 days of age, the four subgroups, each consisting of 10 lambs, received the same diets ad libitum (120L) or restricted (120R) at 75% of the ad libitum intake recorded for the 120L subgroups in the previous week. In this phase, from 90 to 120 days of age, each subgroup was housed under the same conditions as those described for the previous phase. At the end of this period, all the lambs were slaughtered, with the exception of three lambs (one 120R female fed DWB20 diet, and two 120L males, one DWB0 and the other DWB20) that were removed from the experiment due to their poor health status not attributable to feeding treatments.

On the basis of this experimental design, the factors involved were feeding plan (90L, 120R, 120L), defined by the feeding level, ad libitum (L) or restricted at 75% (R), age to slaughter, 90 or 120 days, and diet, without or with 20% DWB in the concentrate (DWB0 and DWB20, respectively); accordingly, the six experimental groups were as follows: DWB0-90L (n = 14), DWB20-90L (n = 14), DWB0-120R (n = 10), DWB20-120R (n = 9), DWB0-120L (n = 9), DWB20-120L (n = 9).

The lambs were managed during the experiment according to the European Directive 2010/63/EU on the protection of animals used for scientific purposes, complying with the Italian Legislative Decree 26/2014, and were slaughtered according to the European Union Regulations (Council Directive 93/119 EEC) on the protection of animals at the time of slaughter or killing.

### 2.2. Measurements, Sampling and Analysis

#### 2.2.1. Feeds

During the experimental period, the weight of feeds offered to lambs from each pen and those refused by the same lamb group were recorded daily to calculate the group feed intake. Samples of the offered feeds (alfalfa hay, faba bean, barley and DWB) were collected every two weeks and analyzed following AOAC [23] methods to determine dry matter (DM) (method 934.01), ether extract (EE) (method 920.39), crude protein (CP) (method 2001.11), ash (method 942.05) and fibrous fractions, as aNDFom (neutral detergent fiber using heat-stable amylase and exclusive of residual ash) (method 2002.04), ADFom (ash free acid detergent fiber) (method 973.18), and ADL (acid detergent lignin) (method 973.18). Nonfiber carbohydrates content was calculated as 100 – (% EE + % CP + % ash + % aNDFom). Equations from the National Research Council were used to estimate the net energy for gain of the feeds (Mcal/kg DM) [24].

Samples of concentrates and their ingredients were also analysed in triplicate for the content of ferulic acid and total phenolic acids, expressed in μg/g DM. Extraction of phenolic acids from feeds samples (250 mg) was processed according to the procedure reported by Laddomada et al. [25], which consisted of, in order, sample delipidation, alkaline hydrolysis, acidification and recovery in ethyl acetate; then, the extracts were dried under nitrogen flux, re-dissolved in 200 µL of 80:20 methanol/water, and analysed in an Agilent 1100 Series HPLC-DAD system (Agilent Technologies, Santa Clara, CA, USA). Separation and identification of peaks were performed as described by Bonanno et al. [20].

Feeds extracts were also prepared in duplicate according to López-Andrés et al. [26] and used to measure the total polyphenol content (mg gallic acid equivalent (GAE)/kg DM) by the Folin-Ciocalteau colorimetric method [27], and the trolox equivalent antioxidant capacity (TEAC, mmol trolox equivalent/kg DM) as described by Re et al. [28].

The fatty acids (FA) of feeds extracted from 50 mg samples followed the one-step extraction and transesterification procedure [29], with C23:0 as the internal standard (Sigma-Aldrich, Milano, Italy), and were identified using the procedure described below for meat FA.

#### 2.2.2. Lambs, Slaughter and Carcasses Assessment

During the entire experiment, lambs were regularly weighed at two-week intervals to evaluate the rate of growth and the feed conversion.

The lambs were weighed at the end of the experiment, then kept fasting from solids for 12 h, after which they were transported to a commercial slaughterhouse and weighed again before slaughter. 

After removing skin, feet and gastrointestinal tract, the hot carcasses were kept at room temperature (>10 °C) for 6 h, chilled to 4 °C for 24 h, and then weighed. The weight of gastrointestinal content was estimated by weighing the full and empty gastrointestinal tract and used for calculation of empty body weight. Afterwards, the chilled carcasses were dissected to separate and weigh the head, internal organs and the right half of the carcass. The perirenal and pelvic fat, hind leg and Longissimus dorsi (LD) muscle were removed from the right half of carcass and then weighed. The hind leg was dissected in its tissue components (lean meat, fat and bone) to determine their incidence and the meat-to-bone ratio. The LD muscle was cut into three parts: proximal (from 8th to 13th vertebra, Thoracis prossimale muscle), intermediate (Thoracis distal muscle) and distal (Longissimus lumborum muscle).

#### 2.2.3. Physical, Chemical and Sensorial Analysis of Meat

The pH was measured on intermediate LD samples 24 h after slaughter (ultimate pH), using a Hanna FC 200 pHmeter equipped with a penetrating probe (Hanna Instruments, Baranzate, Milan, Italy). 

A colorimetric analysis was performed in duplicate on the section of the intermediate LD samples and the perirenal fat after 1 h of exposition at ambient temperature, with a Minolta Chroma Meter CR300 (Minolta corporation, Ltd., Osaka, Japan) using the illuminant C. Results are expressed as lightness (L*, from 0 = black, to 100 = white), redness (a*, from red = +a, to green = −a), and yellowness (b*, from yellow = +b, to blue = −b), according to the CIE L* a* b* system [30]. Chroma (colour intensity or saturation, C = 0 = gray) was calculated as (C = (a* ^2^ + b* ^2^) × 0.5), and hue angle (colour tone, H = 0° = purple red) was calculated as (H = arctg b*/a*) [31].

The LD samples were vacuum packed and frozen at −20 °C for subsequent analysis. The weights of the frozen intermediate LD samples and the corresponding meat thawed at 4 °C for 24 h were used to determine thawing loss. Cooking loss was measured on the same intermediate LD samples wrapped in polyethylene bags, cooked in a water bath at 75 °C for 40 min, cooled for 1 h, and then reweighed to determine moisture loss.

The Warner-Bratzler (WB) shear force was measured on three cylinders of cooked meat with a 12.7 mm diameter using an Instron 5564 (Instron, Trezzano sul Naviglio, Milan, Italy).

After being frozen, the proximal LD samples were freeze-dried and ground and then analysed to determine moisture, fat and ash content according to the AOAC methods [23]. Protein was calculated by difference (100 — % moisture — % fat — % ash).

Lyophilized samples of LD meat of 36 male lambs were used to prepare the aqueous extracts in duplicate according to the procedure described by Luciano et al. [21] to measure the total polyphenols content (mg GAE/kg DM) by the Folin-Ciocalteau colorimetric method [27], and the antioxidant status by TEAC assay (mmol trolox equivalent/kg DM) [28]. 

The fatty acid (FA) composition was determined on lyophilized LD meat from 120-day-old male lambs (n = 18). The extraction of the fat and the preparation of the FA methyl esters (FAME) were performed according to O’Fallon et al. [32]. Briefly, 1 g of sample was hydrolyzed (with KOH in methanol) and methylated (by H_2_SO_4_ catalysis) directly. The FAME were recovered in 1.5 mL hexane and 1 μL of each sample was injected by auto-sampler into an HP 6890 gas chromatography system equipped with a flame ionization detector (Agilent Technologies, Santa Clara, CA, USA). Separation of FAME was performed using a capillary column 100 m in length with an internal diameter of 0.25 mm and film thickness of 0.25 μm (CP-Sil 88, Chrompack, Middelburg, The Netherlands). Gas chromatography conditions and identification of each FA were as described by Bonanno et al. [33]. Each individual FA was expressed as g/100 g total detected FA. The thrombogenic index (TI) was calculated according to Ulbricht and Southgate [34] as follows: TI = (C14:0 + C16:0 + C18:0)/(0.5 × monounsaturated FA (MUFA) + 0.5 × n-6 polyunsaturated FA (PUFA) + 3 × n-3 PUFA + n-3 /n-6). The health-promoting index (HPI) was calculated as proposed by Chen et al. [35]: HPI = (n-3 PUFA + n-6 PUFA + MUFA)/(C12:0 + 4 × C14:0 + C16:0). Finally, the hypocholesterolemic FA/hypercholesterolemic FA ratios (HH) was calculated as suggested by Santos-Silva et al. [36]: HH = (C18:1 c9 + C18:2 n-6 + C20:4 n-6 + C18:3 n-3 + C20:5 n-3 + C22:5 n-3 + C22:6 n-3)/(C14:0 + 16:0).

For the sensory analysis, 12 untrained panelists in two sessions assessed the cooked meat of the distal LD muscle by triangle difference tests, according to Napolitano et al. [37]. The meat was cooked on an electric grill and offered hot. Each panelist was invited to identify the sample that was different from the other two samples. The three samples were presented to panelists in the following 5 triangular combinations: DWB0-90L vs. DWB20-90L; DWB0-120R vs. DWB20-120R; DWB0-120L vs. DWB20-120L; DWB0-120R vs. DWB0-120L; DWB20-120R vs. DWB20-120L. During the same sessions, the assessors were asked to indicate the degree of difference perceived.

### 2.3. Statistical Analysis

Data were processed using the generalized linear model (GLM) procedure of SAS 9.2 software [38].

Parameters related to in vivo performance of lambs were analysed separately for the two growth periods, from 45 to 90 and from 90 to 120 days of age. The daily feed intake recorded for the lambs of each subgroup was analyzed with a model that for the 45–90-day period included the effect of the diet (2 levels: DWB0 and DWB20), and for the 90–120-day period included the effects of feeding level (FL, 2 levels: 120R and 120L), diet and the interaction FL × diet. The individual growth parameters of lambs were analysed with the same models, including the effect of sex as well.

The post-mortem parameters related to slaughter performance and carcass and meat assessment were statistically processed according to a model comprising the feeding plan (FP, 3 levels: 90L, 120R and 120L), diet, sex and the interaction FP × diet. For total polyphenols, TEAC, and FA composition; the model did not include the effect of sex.

Comparisons between least-square means were performed by Tukey’s test when the effects were significant (*p* ≤ 0.05). *P*earson’s coefficients were calculated to test correlation between parameters. In the sensory triangle tests, the significance of differences was assessed using the standard references tables from Amerine et al. [39].

## 3. Results and Discussion

### 3.1. Diets Composition

Table 1 reports the chemical composition, phenolic compounds content, antioxidant activity, and FA profile of the diet ingredients and the experimental concentrates.

The formulation of the concentrates shows that to balance their energy content, the inclusion of 20% of DWB required the reduction of faba bean and barley by 8 and 12 percentage points, respectively. However, the presence of DWB, which is the most fibrous component, resulted in a higher level of aNDFom (+ 5%). In addition, the DWB contributed to double the content of total phenolic acids, consisting largely of ferulic acid, and therefore, the total polyphenols. Thus, the greater antioxidant capacity (TEAC) detected for the DWB20 concentrate can be certainly attributed to the higher presence of phenolic compounds in the bran, especially ferulic acid, which is recognized for its antioxidant power [19]. With regard to the FA composition of concentrates, the DWB was particularly responsible for the increase in unsaturated FA, especially linoleic acid (LA, C18:2 n-6)

### 3.2. Lamb in Vivo Performance

#### 3.2.1. Growth and Feed Intake from 45 to 90 Days of Age

The growth performance and feed intake of lambs during the first phase, from 45 to 90 days of age, are shown in Table 2. Neither the diet nor the sex influenced the growth rate of the animals, and the consequent final body weight at 90 days. Although the lambs fed with DWB showed a higher DM intake from both alfalfa hay and concentrate, these differences did not reach statistical significance; as a consequence, the diet did not significantly affect the concentrate and diet conversion ratios. For both diets, the incidence of concentrate reached a quite high level, around 74%. As expected, with the DWB20 diet, there was an increasing intake of ferulic acid and total phenolic acids.

#### 3.2.2. Growth and Feed Intake from 90 to 120 Days of Age

Table 3 shows the growth performance and feed intake of lambs in the second phase, from 90 to 120 days of age, during which the diets were offered restricted at 75% or ad libitum.

The considered factors significantly affected the weight gain of lambs, which strongly decreased when the DWB-based diet was offered at a restricted level; since the feed intake did not differ between the restricted DWB0 and the DWB20 lambs, this result is certainly attributable to the higher fiber content of the DWB20 concentrate which, reducing the digestive utilization, did not allow the restricted lambs to satisfy their nutritional needs for growth. In addition, in this more advanced phase, when the first signs of sexual dimorphism begin to emerge, the effect of sex on weight gain from 90 to 120 days was almost significant (*p* = 0.052) due to the faster growth rate of males compared to females. These effects were responsible for lower final body weights in lambs fed restricted DWB20 diet and in females, although the differences were not significant.

The DM ingestion of hay and concentrate, as well as the incidence of concentrate in the diet were obviously influenced by the feeding level, whereas no effect of the DWB in the diet emerged. The feed restriction achieved was more accentuated for hay in DWB0 (57%) than in DWB20 diet (70%), and the same (73%) for concentrates. Overall, the restriction in both DWB0 (69%) and DWB20 diets (72%) has a more pronounced result than that planned at 75% of the ad libitum intake. As expected, the ferulic acid and the total phenolic acids were ingested at highest levels with the diet containing DWB offered ad libitum, followed by the restricted DWB20 diet.

Due to the lower weight gain, the levels of the concentrate and diet conversion ratios were particularly high in the lambs fed with the restricted DWB20 diet; indeed, the interaction FL × diet, which expressed a different trend of diets within the feeding levels, tended towards statistical significance. Instead, the feed conversion ratios were significantly higher in the females, which, being characterized by a more precocious adipogenesis compared to males, require more energy to develop adipose tissue which, as known, increases the feed conversion ratio.

The growth rate of lambs recorded from 90 to 120 days of age was comparable to that equal to 178 g/day detected in male lambs of Comisana breed fed ad libitum from 80 to 130 days of age with an analogous diet consisting of alfalfa pelleted hay and a concentrate composed of faba bean (76%) and barley (24%), while the feed conversion ratio was markedly higher than that recorded for the same Comisana lambs, which was equal to 4.82 [40]. However, the growth rates recorded here for lambs of the Valle del Belice dairy breed were markedly lower than those recorded in lambs of breeds with an aptitude for meat production, such as Barbaresca lambs at an age of about 100 days (from 218 to 250 g/day) [41] or 130 days (from 225 to 285 g/day) [42], as well as Fabrianese lambs at 5 months of age (244 g/day) [3].

### 3.3. Lambs’ Slaughter Performance and Carcass Traits

The parameters related to slaughter performance and carcass traits are reported in Table 4.

The feeding plan, defined by different slaughter age of lambs and feeding level, affected significantly the weight of lambs at slaughter; indeed, both the slaughter body weight (22.9 vs. 26.3 and 27.9 kg for 90L, 120R and 120L lambs, respectively) and empty body weight (20.1 vs. 23.1 and 24.8 for 90L, 120R and 120L lambs, respectively) were obviously higher in 120 day-old lambs, especially when fed ad libitum, although the 120L and 120R plans did not have statistically dissimilar results. As a consequence, the weight of the entire (12.5 vs. 14.2 and 15.2 kg for 90L, 120R and 120L lambs, respectively) and half carcasses (5.05 vs. 5.74 and 6.16 kg for 90L, 120R and 120L lambs, respectively) were significantly lower for the younger lambs in comparison with the 120-day-old lambs. On average, the carcasses of 90L lambs weighed 12.5 kg, which is heavier than the carcasses produced from 130-day-old Comisana lambs fed with an analogous diet (11.3 kg) [40], and comparable to those of 100-day Comisana lambs fed green forage of sulla (12.5 kg) [43], and those of 100-day lambs of Barbaresca breed (from 12.1 to 13.1 kg) [41]. Then, during the period from 90 to 120 days, the carcass weight increased on average by 1.7 kg and 2.7 kg with restricted and ad libitum feeding, respectively. On the basis of the carcass classification systems in the European Union (EEC 2137/92 and 461/93 regulations), the 90L carcasses were classified into class C (10.1–13 kg) with quality 2 (red meat or fatness score 1 or 4) of the scheme for lamb carcasses lighter than 13 kg [44], whereas the 120-day carcasses were classified using the scheme for lamb carcasses weighing more than 13 kg and mainly scored as O (fair) for conformation and 2 (slight) for degree of fat cover (most of 120R carcasses) or as R (good) for conformation and 2 (slight) or 3 (average) for degree of fat cover (most of 120L carcasses).

Compared to the 120L lambs, the 90L lambs showed a higher incidence of head (8.11% vs. 7.57 %), a lower presence of perirenal and pelvic fat (2.15% vs. 2.68 %), and a tendency towards a lower incidence of the internal organs. These results can be attributed to the different growth rate of these body regions [45], which is earlier for the head, due to its greater bone base, and later for the fat and internal organs; thus, the head showed a higher percentage in younger lambs, whereas the proportions of fat and organs increased in older lambs.

A tendency towards significance of the feeding plan also emerged for the incidence of the empty gastrointestinal tract and its content, which slightly increased in 120R lambs, and can be explained by the slower gastrointestinal transit associated to restricted feeding that would favor a longer permanence of ingested feeds in the digestive tract and, as a consequence, a slight increase recorded in its volume and content.

Regardless of the feeding plan, the carcasses of lambs fed DWB showed a greater incidence of head, which, also in this case, can be related to the greater development of the more precocious bone tissue [45], favored by restricted or low energy diets. The DWB-based diet was also responsible for lower carcass yields for which, however, emerged a significant FP × diet interaction; indeed, the diet with DWB significantly reduced the carcass yields only in the restricted 120-day-old lambs. This result can be linked mainly to the higher incidences, although at a not significant level, of the empty gastrointestinal tract (+1.2%) and its contents (+1.2%) recorded in DWB20-120R than in DWB0-120R lambs, presumably due to the previously mentioned effect of restriction associated to a higher volume of the more fibrous DWB20 concentrate.

The sex of the lambs influenced the carcass yield expressed as a percentage of the slaughter body weight (SBW), which was higher in the females due to only their lower gastrointestinal content, since the same difference did not emerge for the carcass yield referred to the empty body weight (EBW). The carcasses of the females also showed a greater adiposity, as indicated by the higher content of perirenal and pelvic fat. In contrast, the males had a higher incidence of head, which denotes their greater skeletal development compared to the females. These expected results are in line with several findings on lambs from various breeds and weight ranges [46] and are linked to the earlier maturity of females in comparison with males of same age; the precocity of female lambs is reflected in their greater tendency for fat deposition and lower incidence of bone and muscle tissue, and to the greater nitrogen retention of males, which develop more muscle than adipose tissue.

With regard to the tissue composition (Table 4) that resulted from the right hind leg dissection, the feeding plan affected the weight of the hind leg, which increased passing from 90L to 120R and 120L lambs (1.43 vs. 1.92 and 2.02 kg), and the fat tissue incidence, which also increased in older lambs (5.63 vs. 8.62 vs. 11.28 % in 90L, 120R and 120L lambs, respectively). However, the increasing adiposity recorded in the hind leg of older lambs corresponded to a degree of fat cover scored as 2 (light) or 3 (average), which are the levels denoting lean carcasses. The meat incidence showed an opposite trend due to the feeding plan (66.9 and 65.3 vs. 62.6 % in 90L, 120R and 120L lambs, respectively), whereas no effect emerged for the bone incidence and the meat-to-bone ratio. The diet did not modify these parameters, whereas the sex of lambs influenced the fat and bone incidences; indeed, the hind leg of male lambs was higher in bone and lower in fat, in line with the incidences of head and the perirenal and pelvic fat mentioned previously.

### 3.4. Meat Evaluation

Similarly to the carcass traits, the parameters of physical quality of LD meat were also more affected by the feeding plan rather than by the other factors considered (Table 5); nevertheless, these traits appeared to be mainly influenced by the slaughter age of lambs rather than by the feeding level to the 120-day-old lambs were submitted.

The meat of the lighter carcasses from the 90L lambs showed an ultimate pH (6.01), measured at 24 h after slaughtering, higher than that of meat from 120-day lambs (5.71 and 5.68 for 120R and 120L lambs, respectively). As known, a higher meat pH is linked to a lower post mortem acidification, which, in turn, could depend on the mobilization of the muscle reserves of glycogen to sustain an increasing energy demand [5,47]; in this study, the higher energy expenditure of the 90L lambs could be linked more presumably to the emotional stress induced by the pre-slaughter condition, due to fasting and transport, to which the younger animals could be more susceptible, rather than to the feeding regimen.

Despite its higher pH, which should be linked to an increasing water holding capacity [43], the LD meat of 90L lambs showed greater thawing losses (7.95% vs. 5.52% and 5.96% for 90L, 120R and 120L lambs, respectively), whereas no differences emerged for the water losses due to the successive cooking. The lower water retention observed for the meat of 90L lambs is in agreement with Budimir et al. [9] and Russo et al. [44], who found a lower water holding capacity for lighter carcasses due to a higher drip loss, explained by the weaker ability of myofibrillar proteins of meat from younger lambs to hold water [9].

The higher values of shear force recorded for the 90L cooked meat indicates a lower tenderness of meat from young lambs, especially in comparison with the 120R meat (4.09 vs. 2.65 kg/cm^2^). This result is in line with D’Alessandro et al. [5], who detected a higher collagen content, the main determinant of meat toughness, in the intramuscular connective tissue of younger lambs. Nevertheless, the same authors [5] suggested that collagen shows low variation with age and also weak correlations with the toughness of cooked meat. Accordingly, in this study, the greater water losses after thawing could also have contributed to the lower tenderness of the 90L cooked meat.

The colorimetric parameters of both meat and fat showed differences between the 90L carcasses and those from the 120-day-old lambs. On the whole, the meat of older lambs was brighter and had a more intense red color, as indicated by the higher values of lightness, redness, and chroma. In contrast, the fat of lighter carcasses was less bright and with a more intense color, as expressed by the lower values of lightness and the higher redness, yellowness and chroma.

Color is the main attribute used to appreciate meat freshness, and consumers tend to prefer a pale or pink color for light lamb meat [5], and accept darker meat from older lambs [3]. In line with this study, other authors [3,7,9] found an increased redness, related to the higher content of myoglobin [1], in meat from older and heavier lambs. The levels of red color of meat from Valle del Belice lambs were within the commonly detected ranges; in this regard, the redness of the 90L meat was slightly higher than that observed in 100-day-old Comisana lambs [43], and comparable to that found in Barbaresca lambs of both 100 [41] and 130 days of age [42], and in 5-month-old Fabrianese lambs [3], whereas the darker meat from 120-day-old Valle del Belice lambs approached the redness values observed in 130-day-old Comisana lambs [40] and also in 60-day-old Bergamasca light lambs [9].

The diet only influenced the lightness of fat, which increased with the DWB20 diet, presumably as a consequence of the transfer and deposition to the fat tissue of carotenoid pigments contained in the DWB, including lutein, β-cryptoxanthin, zeaxanthin and β-carotene [48].

No significant FP × diet interaction emerged, while sex affected the pH of meat, which was higher in females at a negligible level, and showed only tendencies towards a higher lightness and a lower chroma of fat in the carcasses of females.

Table 5 reports the chemical composition, polyphenols content and antioxidant capacity of LD meat.

The chemical composition of LD meat was not affected by the feeding plan, in accordance with studies in which the slaughter age or carcass weight did not influence the chemical composition of lamb meat [5,9]. Accordingly, the intramuscular fat content, which represents the later fat deposition, did not increase from 90L to 120R and 120L meat, as occurred for the incidence of earlier fat depots, such as perirenal and pelvic fat in the carcasses and the separable fat tissue in the hind leg. Thus, when prolonging the growth period by 30 days, the lamb meat did not show differences in fat infiltration. In contrast, the diet with DWB reduced the fat content of meat, balanced by an increase of protein, although only at a tendency level. This result indicates the effect of DWB inclusion in the concentrate in reducing the level of intramuscular fat, which emerged with both a restricted and an ad libitum feeding level. Moreover, the calculated amount of total lipid in fresh meat obtained with the DWB inclusion (5.60% and 6.26% with DWB20-120R and DWB20-120L diet, respectively) only was slightly higher than the levels (<5%), indicating lean meat, according to the Food Advisory Committee [49].

The sex of lambs strongly influenced the composition of the LD meat; indeed, the females, due to their earlier maturity, showed a more consistent intramuscular fat deposition to which the reduction of the other components corresponded. 

The content of total polyphenols and the antioxidant capacity of LD meat, detected exclusively for the male lambs, were strongly correlated (r = 0.64; *p* < 0.0001), and were both influenced by feeding plan, diet and their interaction. The significance of the interaction shows that a higher presence of polyphenols in the meat, and the consequent improvement of the meat antioxidant capacity, occurred only in the 120-day-old lambs fed with DWB, regardless of the feeding level. Presumably, this result can be linked to the higher DWB intake of 120-day lambs, but also to a longer accumulation in the tissues of the compounds with antioxidant activity contained in the ingested DWB, such as phenolic acids, especially ferulic acid [19], together with carotenoids [48]. Therefore, the inclusion of DWB in the diet contributed advantageously to increasing the content of phenolic compounds and the antioxidant activity of lamb meat. This result further confirms that ingested polyphenols could move into the muscles, as suggested by Moñino et al. [50], who observed a great presence of polyphenols in the meat of lambs suckling from ewes fed with rosemary extracts. On the other hand, the study of Soberon et al. [51] provided evidence of the possible tissue uptake of free ferulic acid dosed orally in lambs. A higher polyphenol content and antioxidant capacity were also detected in cheeses obtained from milk of cows fed a diet supplemented with 3 kg/day of DWB [20]. Accordingly, the DWB seems to have the potential to enrich the meat of bioactive compounds, consisting of phenolic acids and carotenoids, which are able to improve its oxidative stability and health properties.

In the triangle tests, the panelists were able to detect sensorial differences between samples of cooked meat due to the presence of DWB in the diet and the feeding level, restricted or ad libitum, although these differences were always perceived at a moderate level. In particular, the assessors discriminated significantly the diet in the meat of 90L (DWB0-90L vs. DWB20-90L, 66.7% correct answers, *p* < 0.05) and 120R lambs (DWB0-120R vs. DWB20-120R, 66.7% correct answers, *p* < 0.05), but not in the meat of 120L lambs (DWB0-120L vs. DWB20-120L, 33.3% correct answers). Instead, the effect of the feeding level was perceived significantly only in the meat from lambs fed the diet without the DWB (DWB0-120R vs. DWB0-120L, 83.3% correct answers, *p* < 0.001), and not in the meat from lambs fed the DWB20 diet (DWB20-120R vs. DWB20-120L, 50% correct answers).

### 3.5. Meat Fatty Acid Profile

The FA composition of intramuscular fat of LD meat samples taken from 120-day-old male lambs (Table 6) was mainly influenced by the presence of DWB in the diet, whereas the significant effect of the feeding level emerged especially in the interaction with the diet.

Oleic acid (OA, C18:1 *c9*), recognized for its hypolipidemic effect, which is important for human health to reduce plasma cholesterol and triglycerides [52], has been confirmed as the prevalent FA in lamb meat [2,3]. In this study, the OA was not affected by the feeding treatments; since the most common FA in the dietary components was linoleic acid (LA, C18:2 n-6) (Table 1), the OA derived by the endogenous desaturation of stearic acid originated, in turn, from the biohydrogenation of mainly LA in the rumen [2,40,53].

With regard to the effect of DWB, the DWB20 diet resulted in a decrease of n-6 FA, especially due to the reduction of LA and arachidonic acid (AA), and an increase of n-3 FA for the main contribution of the eicosapentaenoic acid (EPA); these results corresponded to the favourable strong reduction of the n-6/n-3 ratio to values strictly close to the threshold (≤5) recommended by the FAO/WHO [54] in the human diet for the prevention and treatment of chronic diseases. AA and long-chain n-3 FA, especially EPA and docosahexaenoic acid (DHA), are of interest for infant nutrition, as they are essential for optimal neonatal growth and development; in this regard, it can be noticed that their levels recorded with the DWB20-120L were strictly close to those that Nudda et al. [55] found in fresh meat from suckling lambs, which were higher than those detected in commercial lamb-based infant foods.

However, EPA and DHA are recognized for their multiple health benefits, mediated by their anti-inflammatory actions [56,57] and especially for their role in reducing the risk of cardiovascular disorders in humans [58]. Therefore, their presence should encourage lamb meat consumption to reach adequate intakes and reduce the n-6/n-3 ratio of the diet.

On the contrary, the diets with DWB were responsible for the tendency for a lower level of rumenic acid (RA), the main isomer of the conjugated linoleic acids (CLA), known for its health benefits [59,60]; this effect occurred independently of the feeding level, and despite the precursor of the RA, the *trans* vaccenic acid (VA) only showed a reduction with the restricted DWB20 diet.

The VA is an intermediate of the biohydrogenation of dietary polyunsaturated FA to stearic acid performed by the micro-organisms in the rumen, whereas the RA is produced by the endogenous desaturation of VA in the tissues due to the Δ9-desaturase [61]. Thus, the reduction of both VA and RA recorded with DWB-based diets, could be linked to a favourable effect towards a the complete biohydrogenation process in the rumen, more pronounced with lower intakes, as occurred with the restricted level, and confirmed by the higher incidence of stearic acid in the meat fat with the DWB20-120R diet. In this regard, an adverse effect of DWB or its phenolic compounds on the successive VA desaturation can be excluded by the comparable values of the desaturase index of VA obtained among feeding treatments (Table 6).

Significant interactions with the FL × diet emerged for the total of saturated FA (SFA) and unsaturated FA (UFA), and then for their PUFA/SFA and UFA/SFA ratios. The effect of DWB on the level of saturated FA was opposite in relation to the feeding level, since the byproduct induced a decrease of saturated FA, especially palmitic (C16:0) and stearic (C18:0) acids, when offered ad libitum. This result could be a confirmation of the effect of restricted DWB20 diet in favouring the complete biohydrogenation process in the rumen. The levels of saturated FA with DWB20-120L and DWB0-120R diets (37–38 g/100 g FA) resulting from this study were lower than those recorded in meat from light lambs of the Leccese breed (from 50 to 55 g/100 g FA) [2,4] and the Bergamasca breed (51–53 g/100 g FA) [9], and comparable to those of heavier 130-day-old Comisana lambs (36–39 g/100 g FA) [40] and Fabrianese lambs of 2 and 5 months of age (40–42 g/100 g FA) [3]. These comparisons are in agreement with the increase in the unsaturation of fat depots observed with increasing age at slaughter [2].

Compared to the DWB0-120L diet, the DWB20 diet offered ad libitum was responsible for the rise in unsaturated FA, which occurred as a consequence of the reduced saturated FA, and despite the reduction of α-linolenic acid (ALA). Since ALA represents the precursor of the long-chain n-3 FA, its reduction could be linked to the major biosynthesis of EPA, DPA and DHA that emerged with the DWB20-120L diet. Significant interactions also emerged for the health indexes used to assess the health value of meat fat; indeed, when the lambs were fed the DWB20 diet ad libitum, the trombogenic index and the h/H ratio improved by decreasing, whereas the health promoting did not decrease, as occurred with the DWB20 restricted diet.

Moreover, the DWB20-120L diet led to improvements in the PUFA/SFA and UFA/SFA ratios, approaching the level recorded with the DWB0-120R. In particular, the ratio PUFA/SFA of both DWB0-120R and DWB20-120L diets reached closer values to that recommended for human health (0.45) [62].

On the whole, these results show that the presence of DWB in the diets of lambs increased the level on n-3 FA in the meat, especially EPA and DHA, reducing the n-6/n-3 ratio; moreover, when the DWB was offered in a greater amount, as obtained when the DWB20 concentrate was fed ad libitum, the byproduct was able to further improve the health profile of meat FA by reducing the saturated FA, and increasing the more beneficial unsaturated FA and the PUFA/SFA and UFA/SFA ratios.

## 4. Conclusions

The feeding plans proposed in this investigation, consisting of a feed restriction at 75% applied after 90 days of age (DWB0-120R), or based on the inclusion of DWB as a fibrous source in a diet offered constantly ad libitum (DWB20-120L), were both suitable to increase the slaughter age of dairy breed lambs to up to 120 days, and produce lean carcasses of about 15 kg, which are heavier than those of traditional milk-fed lambs. Regardless of diet, the quality of meat from 120-day lambs, compared to that from 90-day lambs, improved in terms of major water retention, tenderness and lightness. The use of DWB reduced the fat level of 120-day lamb meat and improved its health properties by increasing the polyphenols content, the antioxidant capacity and the level of n-3 polyunsaturated FA. However, the use of DWB associated to feed restriction is not recommended, since it resulted in a lowest growth performance and carcasses weight of 120-day lambs.

Ultimately, the results obtained applying the DWB0-120R or DWB20-120L feeding plan were analogous in terms of lambs’ growth rate, feed conversion and carcass weight. Thus, the choice between them depends on the preferred method to limit feeding costs, which can be obtained by reducing feed intake by 30% due to the feed restriction, or by using a less expensive feeding source, such as the DWB, but also taking into account the benefits linked to the use of the DWB in terms of environmental sustainability, the oxidative stability of meat and protection of consumers’ health.

## Figures and Tables

**Table 1:** Formulation of the concentrates, and chemical composition, antioxidant capacity and fatty acids of the diet ingredients and concentrates.

Items	Ingredients				Concentrates	
	Alfalfa Hay	Faba Bean Grains	Barley Grains	Durum Wheat Bran	DWB0	DWB20
Faba bean grains, %					66	58
Barley grains, %					34	22
Durum wheat bran, %						20
						
Dry matter (DM), %	90.4	87.8	90.6	87.9	88.7	88.4
Ether extract, % DM	1.67	1.55	2.11	5.64	1.77	2.38
Crude protein, % DM	9.16	29.2	10.0	16.9	23.2	22.1
Ash, % DM	11.5	3.80	2.98	5.06	3.96	3.85
Non-structural carbohydrates, % DM	13.1	46.6	64.0	35.5	52.4	48.0
aNDFom, % DM	64.6	18.4	20.4	36.9	18.7	23.6
ADFom, % DM	52.7	13.1	10.6	13.1	12.3	12.2
ADL, % DM	9.49	0.91	1.49	3.63	1.18	1.69
Net energy for gain, Mcal/kg DM	0.79	1.99	1.98	1.80	1.97	1.94
Ferulic acid, μg/g DM	-	16.78	799	1954	340	782
Total phenolic acids, μg/g DM	-	36.0	1273	2345	503	1028
Total polyphenols, g GAE/kg DM	3.12	2.51	8.27	12.60	4.40	5.91
TEAC, mmol trolox eq/kg DM	17.0	17.5	70.6	87.5	71.1	96.6
Fatty acids, g/kg DM						
C12:0	0.53	-	0.074	-	0.037	0.022
C14:0	2.80	0.013	0.31	5.51	2.32	2.10
C16:0	2.94	1.85	3.14	7.68	2.46	3.16
C16:1 c9	0.23	0.018	0.051	0.11	0.029	0.039
C18:0	0.54	0.58	0.50	0.42	0.33	0.33
C18:1 c9, OA	2.83	2.02	3.23	8.85	3.13	4.96
C18:2 n-6, LA	3.69	7.20	8.38	27.48	7.48	11.5
C18:3 n-3, ALA	0.76	1.32	0.99	3.09	0.97	1.23

DWB0 and DWB20 = concentrates with 0% and 20% of durum wheat bran. GAE = gallic acid equivalent. TEAC = Trolox equivalent antioxidant capacity. OA = oleic acid. LA = linoleic acid. ALA = α-linolenic acid.

**Table 2:** Growth performance and feed intake of lambs from 45 to 90 days of age.

Items	Diet			Sex			*p*-Value	
	DWB0	DWB20	SEp	Females	Males	SEp	Diet	Sex
Lambs, n.	34	34		30	38			
Initial body weight at 45 days, kg	16.3	16.2	0.47	16.6	15.9	0.67	0.91	0.25
Final weight at 90 days of age, kg	23.8	23.7	0.64	24.1	23.4	0.91	0.88	0.47
Weight gain at 45–90 days, g/day	168	166	6.53	166	168	9.27	0.89	0.89
*Feed intake (g/day per lamb)*								
Alfalfa hay DM	206	244	19.5				0.18	
Concentrate DM	627	710	63.7				0.37	
Diet DM	833	954	78.6				0.29	
Concentrate, % diet	73.7	74.6	1.53				0.68	
Ferulic acid, mg/day	213	555	38.8				<0.0001	
Total phenolic acids, mg/day	316	730	52.0				<0.0001	
Concentrate conversion ratio	4.13	4.44	0.26	4.15	4.41	0.38	0.40	0.48
Diet conversion ratio	5.48	5.96	0.35	5.54	5.90	0.50	0.33	0.48

DWB0, DWB20 = concentrate with 0% or 20% of durum wheat bran. Sex: F = females, M = males. Sep = pooled standard error.

**Table 3:** Growth performance and feed intake of lambs from 90 to 120 days of age.

Feeding Level (FL)	120R		120L			Sex			*p*-Value			
Diet	DWB0	DWB20	DWB0	DWB20	SEp	Females	Males	SEp	FL	Diet	FL × Diet	Sex
Lambs, n.	10	9	9	9		19	18					
Initial body weight at 90 days, kg	23.9	23.4	23.6	24.2	2.35	23.6	24.0	1.17	0.85	0.93	0.65	0.76
Final weight at 120 days of age, kg	29.0	26.6	29.1	29.9	2.52	28.0	29.3	1.26	0.18	0.53	0.22	0.30
Weight gain at 90–120 days, g/day	170 a	105 b	185 a	190 a	32.0	146	179	16.0	0.004	0.07	0.04	0.052
Feed intake, g/day per lamb												
Alfalfa hay DM	235	259	409	371	32.2				<0.0001	0.83	0.34	
Concentrate DM	754	765	1032	1055	48.7				<0.0001	0.73	0.90	
Diet DM	989	1024	1441	1426	75.2				<0.0001	0.89	0.74	
Concentrate, % diet	76.8	75.4	72.0	74.3	1.50				0.06	0.81	0.22	
Ferulic acid, mg/day	256 c	598 b	350 c	825 a	31.0				<0.0001	<0.0001	0.04	
Total phenolic acids, mg/day	379 c	786 b	520 c	1084 a	41.4				<0.0001	<0.0001	0.06	
Concentrate conversion ratio	5.69	7.94	5.86	5.78	1.44	7.06	5.57	0.72	0.17	0.14	0.11	0.047
Diet conversion ratio	7.46	10.6	8.18	7.80	1.91	9.51	7.53	0.98	0.28	0.15	0.07	0.047

120R, 120L = restricted (R) or ad libitum (L) feeding level from 90 to 120 days of lambs’ age. DWB0, DWB20 = concentrate with 0% or 20% of durum wheat bran. Sex: F = females, M = males. Sep = pooled standard error. On rows: a, b, c = *p* ≤ 0.05.

**Table 4:** Slaughter performance of lambs and carcass traits in relation to feeding plan, diet and sex.

Feeding Plan (FP)	90L		120R		120L			Sex			*p*-Value			
Diet	DWB0	DWB20	DWB0	DWB20	DWB0	DWB20	SEp	Females	Males	SEp	FP	Diet	FP × Diet	Sex
Lambs, n.	14	14	10	9	9	9		29	36					
Slaughter body weight (SBW), kg	23.0	22.7	26.4	26.2	28.3	27.6	2.77	25.5	25.9	0.92	<0.0001	0.63	0.97	0.62
Empty body weight (EBW), kg	20.3	19.9	23.4	22.9	25.2	24.5	2.42	22.6	22.8	0.81	<0.0001	0.53	0.99	0.86
Carcass at 24 h (CRC), kg	12.6	12.4	14.7	13.7	15.6	14.9	1.59	14.0	14.0	0.53	0.0002	0.23	0.83	0.98
Carcass yield at 24 h, % SBW	54.6 ^a^	54.4 ^ab^	55.7 ^a^	52.3 ^b^	55.0 ^a^	54.0 ^ab^	1.35	54.9	53.8	0.48	0.54	0.001	0.02	0.02
Carcass yield at 24 h, % EBW	62.0 ^ab^	61.9 ^ab^	63.0 ^a^	60.0 ^b^	61.9 ^ab^	60.7 ^ab^	1.33	61.8	61.3	0.45	0.45	0.002	0.02	0.24
Empty gastrointestinal tract, % SBW	22.4	23.1	23.1	24.3	22.7	22.4	1.39	22.6	23.4	0.46	0.09	0.27	0.45	0.12
Gastrointestinal content, % SBW	11.8	12.2	11.6	12.8	11.1	11.0	1.35	11.3	12.2	0.40	0.06	0.23	0.38	0.01
Head, % CRC	8.08	8.15	7.67	8.29	7.40	7.75	0.48	7.64	8.14	0.16	0.02	0.03	0.33	0.03
Internal organs, % CRC	9.87	9.81	10.7	10.1	10.1	9.99	0.62	10.2	10.1	0.21	0.07	0.23	0.50	0.74
Half carcass (HC), kg	5.10	4.99	5.93	5.54	6.38	5.95	0.66	5.68	5.62	0.23	0.0007	0.19	0.81	0.79
Perirenal and pelvic fat, % HC	2.13	2.17	2.37	2.18	2.66	2.70	0.52	2.78	1.96	0.18	0.04	0.84	0.81	<0.0001
Hind leg (HL), kg	1.44	1.42	2.00	1.85	2.06	1.98	0.22	1.81	1.77	0.076	<0.0001	0.25	0.78	0.59
Meat, % HL	66.4	67.3	65.7	64.8	63.1	62.1	2.01	65.0	64.8	0.67	<0.0001	0.57	0.35	0.77
Fat, % HL	5.82	5.45	8.80	8.43	10.74	11.83	1.78	9.12	7.90	0.59	<0.0001	0.84	0.53	0.04
Bone, % HL	27.8	27.3	25.5	26.8	26.1	26.1	1.92	25.9	27.3	0.65	0.10	0.69	0.46	0.03
Hind leg meat/bone ratio	2.42	2.48	2.61	2.46	2.45	2.39	0.22	2.53	2.41	0.060	0.43	0.52	0.46	0.10

90L = ad libitum (L) feeding level from 45 to 90 days of lambs’ age. 120R, 120L = restricted (R) or ad libitum (L) feeding level from 90 to 120 days of lambs’ age. DWB0, DWB20 = concentrate with 0% or 20% of durum wheat bran. Sex: F = females, M = males. SEp, pooled standard error. On rows: a, b = *p* ≤ 0.05.

**Table 5:** Physical and chemical evaluation of *Longissimus dorsi* (LD) meat and perirenal fat of lambs in relation to feeding plan, diet and sex.

Feeding Plan (FP)	90L		120R		120L			Sex			*p*-Value			
Diet	DWB0	DWB20	DWB0	DWB20	DWB0	DWB20	SEp	Females	Males	SEp	FP	Diet	FP ×diet	Sex
Ultimate pH	6.02	6.01	5.70	5.72	5.70	5.66	0.073	5.83	5.77	0.075	<0.0001	0.79	0.66	0.02
Thawing loss, %	8.11	7.79	6.01	5.03	6.85	5.07	1.92	6.37	6.59	0.64	0.004	0.11	0.63	0.72
Cooking loss, %	15.0	16.4	12.8	10.1	14.3	12.4	5.97	12.9	14.1	1.98	0.19	0.60	0.63	0.52
Total loss, %	21.9	22.9	18.0	14.5	20.3	16.8	6.05	18.4	19.7	2.02	0.04	0.32	0.53	0.52
Shear force on cooked meat, kg/cm^2^	4.15	4.04	2.48	2.82	3.52	3.23	0.77	3.27	3.48	0.25	<0.0001	0.96	0.63	0.43
Meat lightness, L*	40.0	40.4	41.7	45.4	42.1	41.8	3.33	41.2	42.6	1.11	0.04	0.25	0.31	0.21
Meat redness, a*	16.9	17.0	19.9	17.1	19.7	19.3	1.97	18.4	18.2	0.66	0.007	0.11	0.17	0.73
Meat yellowness, b*	4.34	4.62	6.11	4.22	5.82	5.55	1.35	5.01	5.21	0.45	0.09	0.17	0.12	0.67
Meat chroma	17.5	17.7	20.8	17.6	20.6	20.1	2.12	19.1	19.0	0.71	0.007	0.11	0.12	0.86
Meat hue angle	14.3	14.7	17.1	13.7	16.5	15.8	3.58	15.1	15.6	1.19	0.50	0.30	0.39	0.68
Fat lightness, L*	72.1	74.6	76.6	78.7	77.2	77.6	1.85	76.7	75.6	0.62	<0.0001	0.011	0.34	0.08
Fat redness, a*	10.8	10.3	8.35	7.70	6.97	7.02	1.73	8.14	8.93	0.57	<0.0001	0.33	0.89	0.18
Fat yellowness, b*	11.0	11.0	10.3	8.97	8.98	8.93	1.58	9.47	10.3	0.53	0.005	0.37	0.55	0.15
Fat chroma	15.7	15.2	13.4	11.9	11.6	11.6	1.69	12.7	13.7	0.56	<0.0001	0.23	0.57	0.06
Fat hue angle	45.7	46.9	51.0	49.5	51.8	51.9	6.84	49.7	49.2	2.28	0.11	0.97	0.88	0.81
Dry matter (DM), %	26.4	26.1	26.1	25.7	25.7	26.1	1.52	27.0	24.9	0.50	0.77	0.81	0.78	0.0001
Fat, % DM	26.0	25.9	29.0	21.8	27.1	24.0	5.95	29.4	21.9	1.99	0.97	0.09	0.33	0.0003
Protein, % DM	70.0	70.0	67.2	74.0	68.8	71.8	5.61	66.7	73.9	1.87	0.96	0.08	0.31	0.0003
Ash, % DM	4.01	4.07	3.83	4.17	4.08	4.16	0.39	3.85	4.26	0.13	0.78	0.24	0.63	0.003
Total polyphenols ^1^, g GAE/kg DM	0.72 ^b^	0.64 ^b^	1.10 ^b^	1.62 ^a^	1.02 ^b^	1.65 ^a^	0.31				<0.0001	0.001	0.02	
TEAC ^1^, mmol trolox eq/kg DM	10.9 ^bc^	9.69 ^c^	12.8 ^ab^	14.9 ^a^	12.8 ^ab^	15.7 ^a^	1.79				<0.0001	0.04	0.02	

90L = ad libitum (L) feeding level from 45 to 90 days of lambs’ age. 120R, 120L = restricted (R) or ad libitum (L) feeding level from 90 to 120 days of lambs’ age. DWB0, DWB20 = concentrate with 0% or 20% of durum wheat bran. Sex: F = females, M = males. GAE = gallic acid equivalent. TEAC = Trolox equivalent antioxidant capacity. ^1^ On male lambs (n. 36). Sep = pooled standard error. On rows: a, b, c = *p* ≤ 0.05.

**Table 6:** Fatty acid composition (g/100 g FA) of LD meat from 120-day-old male lambs.

Feeding Level (FL)	120R		120L			*p*-Value		
Diet	DWB0	DWB20	DWB0	DWB20	SEp	FL	Diet	FL × Diet
C10:0	0.14	0.37	0.38	0.51	0.10	0.07	0.08	0.59
C12:0	0.34	0.49	0.47	0.79	0.13	0.11	0.08	0.52
C14:0	2.44	2.91	3.01	2.70	0.23	0.45	0.75	0.11
C15:0 *iso*	0.38	0.40	0.18	0.20	0.11	0.09	0.85	0.96
C15:0 *anteiso*	0.52	0.50	0.42	0.54	0.14	0.81	0.71	0.59
C14:1 c9	0.29	0.00	0.35	0.30	0.087	0.048	0.06	0.19
C16:0 *iso*	1.00	0.92	0.88	0.90	0.24	0.78	0.90	0.84
C15:1*cis*	1.06	1.03	0.85	1.05	0.28	0.75	0.77	0.70
C16:0	18.7 ^b^	21.1 ^ab^	22.2 ^a^	19.8 ^ab^	0.65	0.12	0.96	0.001
C17:0 *iso*	0.28 ^a^	0.00 ^b^	0.14 ^ab^	0.27 ^a^	0.077	0.37	0.35	0.02
C17:0 *anteiso*	0.42	0.22	0.48	0.66	0.12	0.051	0.93	0.14
C16:1 c9	2.25	1.48	2.20	2.07	0.25	0.30	0.09	0.21
C17:0	0.69	0.53	0.31	0.85	0.18	0.85	0.29	0.07
C18:0 *iso*	0.78 ^a^	0.00 ^b^	0.00 ^b^	0.30 ^ab^	0.16	0.14	0.14	0.002
C17:1 c9	5.84	5.64	4.89	4.39	0.44	0.02	0.44	0.74
C18:0	12.0 ^b^	13.0 ^a^	11.7 ^b^	10.6 ^c^	0.24	<0.0001	0.93	0.0002
Other C18:1 *trans*	1.44 ^b^	1.98 ^b^	1.67 ^b^	4.15 ^a^	0.45	0.02	0.03	0.04
C18:1 *t11,* VA	3.10 ^a^	1.77 ^b^	2.56 ^a^	2.54 ^a^	0.32	0.72	0.048	0.052
C18:1 c9, OA	30.3	32.5	30.9	30.5	1.75	0.70	0.61	0.47
Other C18:1 *cis*	2.92	1.15	2.50	2.29	0.42	0.40	0.03	0.08
Other C18:2 *trans*	0.74 ^ab^	0.50 ^ab^	0.26 ^b^	1.20 ^a^	0.22	0.62	0.12	0.014
C18:2 n-6, LA	9.34	8.44	8.99	7.91	0.38	0.26	0.02	0.81
C18:3 n-3, ALA	0.82 ^b^	0.95 ^ab^	1.16 ^a^	0.90 ^b^	0.062	0.03	0.29	0.005
CLA C18:2 c9*t11*, RA	0.75	0.62	0.71	0.64	0.049	0.84	0.06	0.56
C20:4 n-6, AA	2.74	1.58	2.56	2.24	0.27	0.39	0.013	0.14
C20:5 n-3, EPA	0.16	0.55	0.27	0.69	0.16	0.44	0.02	0.91
C22:5 n-3, DPA	0.63	0.69	0.00	0.29	0.17	0.008	0.33	0.51
C22:6 n-3, DHA	0.00	0.63	0.00	0.73	0.18	0.77	0.001	0.77
Branched chain FA	3.38	2.03	2.11	2.89	0.67	0.76	0.68	0.13
Saturated FA, SFA	37.2 ^b^	40.1 ^a^	40.0 ^a^	37.9 ^b^	0.34	0.51	0.28	<0.0001
Monounsaturated FA, MUFA	47.6	45.9	46.1	47.5	0.86	0.94	0.91	0.09
Polyunsaturated FA, PUFA	15.2	13.9	13.9	14.6	0.79	0.71	0.72	0.25
Unsaturated FA, UFA	62.8 ^a^	59.9 ^b^	60.0 ^b^	62.1 ^a^	0.34	0.51	0.27	<0.0001
PUFA/SFA	0.41 ^a^	0.35 ^b^	0.35 ^b^	0.39 ^ab^	0.020	0.59	0.60	0.03
UFA/SFA	1.69 ^a^	1.49 ^b^	1.50 ^b^	1.64 ^a^	0.023	0.44	0.25	<0.0001
n-6 PUFA	12.1	10.02	11.6	10.1	0.47	0.67	0.001	0.49
n-3 PUFA	1.61	2.81	1.43	2.61	0.40	0.63	0.007	0.98
n-6/n-3	8.92	5.25	9.04	4.23	1.32	0.74	0.004	0.68
Desaturase ratio RA/VA+RA	0.19	0.29	0.22	0.22	0.08	0.57	0.11	0.11
Thrombogenic index	0.95 ^ab^	1.03 ^b^	1.11 ^a^	0.90 ^b^	0.054	0.86	0.23	0.014
Health-promoting index	2.16 ^a^	1.79 ^b^	1.71 ^b^	1.93 ^ab^	0.083	0.07	0.36	0.002
h/H	2.09 ^a^	1.90 ^bc^	1.75 ^c^	1.93 ^ab^	0.046	0.003	0.94	0.0006

120R, 120L = restricted (R) or ad libitum (L) feeding level from 90 to 120 days of lambs’ age. DWB0, DWB20 = concentrate with 0% or 20% of durum wheat bran. VA = *trans* vaccenic acid. OA = oleic acid. LA = linoleic acid. ALA = α-linolenic acid. CLA = conjugated linoleic acid. RA = rumenic acid. AA = arachidonic acid. EPA = eicosapentaenoic acid. DPA = docosapentaenoic acid. DHA = docosahexaenoic acid. Thrombogenic index = (C14:0 + C16:0 + C18:0)/(0.5 × MUFA + 0.5 × n-6 PUFA + 3 × n-3 PUFA + n-3/n-6) [34]. Health-promoting index = n-3 PUFA + n-6 PUFA + MUFA)/(C12:0 + 4 × C14:0 + C16:0) [35]. h/H = hypocholesterolemic FA/Hypercholesterolemic FA ratios = (C18:1 c9 + C18:2 n-6 + C20:4 n-6 + C18:3 n-3 + C20:5 n-3 + C22:5 n-3 + C22:6 n-3)/(C14:0 + 16:0) [36]. SEp, pooled standard error. On rows: a, b, c = *p* ≤ 0.05.
